# Controlling the Reactivity of the [P_8_W_48_O_184_]^40−^ Inorganic Ring and Its Assembly into POMZite Inorganic Frameworks with Silver Ions

**DOI:** 10.1002/anie.201911170

**Published:** 2019-10-17

**Authors:** Cai‐Hong Zhan, Qi Zheng, De‐Liang Long, Laia Vilà‐Nadal, Leroy Cronin

**Affiliations:** ^1^ School of Chemistry The University of Glasgow Glasgow G12 8QQ UK; ^2^ Key Laboratory of the Ministry of Education for Advanced Catalysis Materials, Institute of Physical Chemistry Zhejiang Normal University Jinhua 321004 China

**Keywords:** co-crystallization, gigantic clusters, molecular growth, polyoxometalates, tungsten

## Abstract

The construction of pure‐inorganic framework materials with well‐defined design rules and building blocks is challenging. In this work, we show how a polyoxometalate cluster with an integrated pore, based on [P_8_W_48_O_184_]^40−^ (abbreviated as {P_8_W_48_}), can be self‐assembled into inorganic frameworks using silver ions, which both enable reactions on the cluster as well as link them together. The {P_8_W_48_} was found to be highly reactive with silver ions resulting in the in situ generation of fragments, forming {P_9_W_63_O_235_} and {P_10_W_66_O_251_} in compound (**1**) where these two clusters co‐crystallize and are connected into a POMZite framework with 11 Ag^+^ ions as linkers located inside clusters and 10 Ag^+^ linking ions situated between clusters. Decreasing both the concentration of Ag^+^ ions, and the reaction temperature compared to the synthesis of compound (**1**), leads to {P_8_W_51_O_196_} in compound **2** where the {P_8_W_48_} clusters are linked to form a new POMZite framework with 9 Ag^+^ ions per formula unit. Further tuning of the reaction conditions yields a cubic porous network compound (**3**) where {P_8_W_48_} clusters as cubic sides are joined by 4 Ag^+^ ions to give a cubic array and no Ag^+^ ions were found inside the clusters.

## Graphical abstract

**Silver is the key**: A polyoxometalate cluster based on the superlacunary heteropolyanion [P_8_W_48_O_184_]^40−^ (abbreviated as {P_8_W_48_}) reacts with silver ions to self‐assemble three new all‐inorganic metal–oxide porous materials that expand the family of zeolitic polyoxometalate frameworks. The silver ions both enable reactions on the clusters as well as link them together.

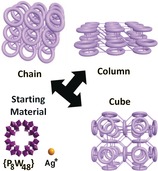

Polyoxometalate (POM)‐based materials are a family of compounds known for their rich structural diversity and properties.^1^ Over the years many synthetic strategies have been developed to control the self‐assembly of the POMs as effective anionic molecular inorganic building blocks.^2^, ^3^ One of the most recent successes of this strategy was made possible by using the superlacunary cyclic heteropolyanion [P_8_W_48_O_184_]^40−^ (abbreviated as {P_8_W_48_}) as a building block for the construction of intrinsically porous all‐inorganic framework materials, named POMzites.^4^ Polyoxometalate‐based framework materials, or POMzites, are an emerging class of configurable all‐inorganic porous materials.^5^ Porous materials such as zeolites or metal–organic frameworks, MOFs, are ordered networks whose building units are linked with strong interactions via ionic, covalent, and coordination bonds.^6^ Silver ions are often used as a flexible linkers in coordination chemistry,^7^ for instance, silver‐linked molybdenum oxide POMs are very stable, both in the solid and liquid phases.^8^ Further, silver ions play a remarkable role in chemical reactions and crystallization processes acting both as countercations and structure directors. In this work, we demonstrate that by using silver ions as linkers, two new porous polyoxotungstates (POTs) can be derived from {P_8_W_48_}, which was isolated originally by Contant and Tezé in 1985.^9^ The {P_8_W_48_} cluster has a symmetry of point group *D*_4*h*_ and is a highly stable and versatile oligomer formed from the aggregation of four subunits of the hexavacant [P_2_W_12_O_48_]^14−^ polyoxoanion derived from the phosphotungstate Dawson‐type cluster [P_2_W_18_O_62_]^6−^.^10^ In 2005, Kortz et al. reported the first Cu‐containing {P_8_W_48_} assembly, thus proving this molecule to be a large superlacunary polyanion precursor.^11^ Since then, a series of novel structures based on {P_8_W_48_} clusters have been reported in the literature including {Cu_20_} clusters;^12^ {V_12_} aggregates;^13^ {Fe_16_}^14^ and Ln‐containing cluster anions;^15^ as well as organoruthenium‐based composites.^16^ All of these structures were based on transition metal complexes with the superlacunary {P_8_W_48_} cluster acting as a ligand.^17^ Herein, we present a unique method of extending the {P_8_W_48_}‐based frameworks to form a higher nuclearity species by binding additional lacunary units, as well as building new POMzites.

The {P_8_W_48_} cluster can be viewed as a drum‐like structure formed by the condensation of four {P_2_W_12_O_48_} subunits. Around the corners of the {P_2_W_12_O_48_} subunits on the top and bottom faces, there are eight sites where {W_1_} units or other transition metal ions can be added to form new locations for further growth to extend the structures. The tungsten occupancy on these new growth points is significant, and can be differentiated from other transition metal ions like Co^2+^ or Mn^2+^.^18^ Although these growth points have been noted as being occupied by both tungsten and other transition metals in previous publications,^16^, ^17^, ^18^, ^20^ no extended structures built upon these foundations have been reported.

Our work demonstrates the formation of higher nuclearity clusters built up from the superlacunary {P_8_W_48_} cluster by extension of these growth sites (Scheme 1). The reaction of {P_8_W_48_} and a high concentration of Ag^+^ ions as starting materials led to the formation of Li_8_K_9.5_Ag_21_[H_16_P_10_W_66_O_251_]_0.5_ [H_14_P_9_W_63_O_235_]_0.5_Cl_2_ **⋅**50 H_2_O (**1**), when an aqueous solution (adjusted to pH 1.53 by concentrated HNO_3_) containing LiNO_3_, K_28_Li_5_[H_7_P_8_W_48_O_184_] and AgNO_3_ was heated at 80 °C for about 30 min. The solution was left to cool to room temperature, and after two weeks well‐defined colorless block crystals started to form in solution; those crystals were harvested after further two weeks. The critical conditions for synthesizing compound (**1**) are: i) heating {P_8_W_48_} in the presence of Ag^+^ ions at high concentration; ii) ensuring the ratio of {P_8_W_48_} to Ag^+^ ions in the synthesis is ca 1:30. A similar procedure without heating, and with a lower concentration of silver ions so the cluster to silver ion ratio is 1:12, gives Li_8_K_13_Ag_13_[H_12_P_8_W_51_O_196_]⋅50 H_2_O (**2**). As is common in POM chemistry, the pH is also a decisive parameter that affects the crystallization process. Under reaction conditions similar to that for (**2**), but with a slightly lowered pH, compound Li_10_K_12_Ag_4_ [H_14_P_8_W_48_O_184_]⋅170 H_2_O (**3**), with a cubic structure, was obtained.

**Scheme 1:**
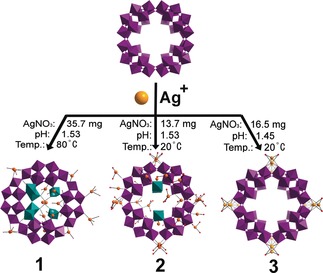
Schematic representation of products depending on reaction conditions and Ag^+^ ions located inside and outside high‐nuclearity clusters produced from the macrocyclic drum‐like [P_8_W_48_O_184_]^40−^ heteropolyanions. Color acheme: W, purple and teal polyhedra; Ag, orange spheres; O, red spheres; Cl, turquoise spheres.

Compound (**1**) crystallizes in a monoclinic system with the space group *P*2_1_/*m*. A {P_8_W_48_} base with six {W_1_} units, at the joining corners between the four {P_2_W_12_O_48_} units, was identified in the structure. An additional lacunary Keggin {PW_9_} or a lacunary Dawson {P_2_W_12_} subunit was found to add on the six {W_1_} units (Figure 1), which are identified as growth points (Figure S1, see the Supporting Information). Since co‐crystallization occurs with these two clusters, the {PW_9_} and {P_2_W_12_} add‐on units co‐occupy the same location by sharing the first six W atoms with full occupancy (Figure S2). The remaining W atom sites that separately belong to the {PW_9_} and {P_2_W_12_} units are 50 % occupied. Most oxo ligands with full occupancies on the {PW_9_} unit are shared by the {P_2_W_12_} unit. The remaining unshared oxo sites with half occupancies on {PW_9_} and {P_2_W_12_} units were also found and refined (Figure S1). A well‐defined disorder model, which includes a 1:1 ratio of {PW_9_} and {P_2_W_12_} units, confirms the co‐crystallization of the {P_9_W_63_O_235_} and {P_10_W_66_O_251_} clusters. Both clusters have the same {P_8_W_48_} base and have roughly similar sizes for the {PW_9_} and {P_2_W_12_} parts. The formation of high‐nuclearity {P_9_W_63_O_235_} and {P_10_W_66_O_251_} clusters is only possible with a high concentration of Ag^+^ ions and heating/refluxing during the synthesis process before crystallization. A careful analysis of the cluster structure found in compound (**1**) revealed that the central cavity of the {P_8_W_48_} base is filled with Ag^+^ ions which form a {Ag_10_} aggregate with two Cl^−^ ions as cores, which is similar to the observation in other Ag‐POM clusters.17a One more Ag^+^ ion was found to support and stabilize key {W_1_} growth sites, similar to the {Ag_10_} cluster, from within the central cavity of the cluster (Figure S2). The remaining 10 Ag^+^ ions per formula were found to link clusters which form a 3D network with a complex topology. The silver ions appear to use all the linking models of {W_48_} that are possible for the POMzite archetypes.^5^ The packing diagram in Figure S3 shows that the space between clusters accommodates silver ions at locations inside and between the clusters.

**Figure 1:**
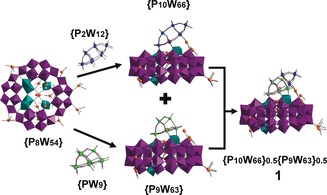
Structural representation of compound (**1**) built from {P_8_W_54_} capped by lacunary {PW_9_} and {P_2_W_12_} units disordered in 1:1 ratio. Color scheme: WO_6_ in {W_48_} units, purple polyhedra; WO_6_ unit on growth points, teal polyhedra; W in {P_2_W_12_} unit, blue spheres; W in {PW_9_} unit, light green spheres; shared W sites of {PW_9_} and {P_2_W_12_} units, teal spheres; Ag, orange spheres; O, red spheres; Cl, turquoise spheres.

Compound Li_8_K_13_Ag_13_[H_12_P_8_W_51_O_196_]⋅50 H_2_O (**2**) was synthesized under reaction conditions similar to (**1**), but at room temperature and with a lower concentration of AgNO_3_. In the single‐crystal structure determination, major silver ion positions are clearly defined with occupancies between 0.55 and 1.0. The structure can be described as {P_8_W_48_} rings that are packed in a layer‐by‐layer mode. Within a single layer, the {P_8_W_48_} rings are virtually coplanar with ring center‐to‐center distances being about 22.9 and 24.2 Å in the two distinct dimensions. Each layer has a thickness of around 11.3 Å. The {P_8_W_48_} rings either within or between layers are linked by Ag^+^ ions (total 9 per formula, Figure S4) and the gaps and cavities are further filled by K^+^ countercations. The closest contact point between clusters is bridged by a Ag^+^ ion with an O⋅⋅⋅O separation of about 2.86 Å. Layers of the {P_8_W_48_} rings are displaced and are stacked in an AB fashion (see Figure 2). There are three additional tungsten atoms with partial occupancies across six growth sites in the {P_8_W_48_} ring in comparison to the above description of (**1**). This confirms the existence of the growth points of the {P_8_W_48_} in solution starting with either a minor tungstate impurity in the starting material, or the decomposition of a small amount of {P_8_W_48_} during the reaction. In contrast to the Ag^+^ cluster found in the cavity of {P_8_W_48_} rings in (**1**), the corresponding positions in (**2**) are instead occupied by K^+^ ions. The network structure of (**3**) is best described as a “truncated cuboctahedron” (or great rhombicuboctahedron); in other words, the molecular paneling of [P_8_W_48_O_184_]^40−^ units into the network can essentially be viewed as cubic (Figure 3), a topology shared by some other notable materials.^19^ Strikingly, this topology is analogous to that of the prominent LTA (Linde Type A) zeolite framework, with a pore size of 0.4 nm.^19^ Both compound (**3**) and POMzite‐3^20^ [Mn_8_(H_2_O)_48_P_8_W_48_O_184_]^24−^ have approximately spherical voids with an internal diameter of ≈2.1 nm (that is, a void volume of ≈4.8 nm^3^, see Figure 3) and are accessible through the integrated pores of the six surrounding {P_8_W_48_O_184_}^40−^ anions.^20^ LTA, compound (**3**), and POMzite‐3 frameworks crystallize in the same cubic *Pm* $\bar{3}$ *m* space group and differ only in the tiling around the central cubo‐octahedral cavity.^19^, ^20^

**Figure 2:**
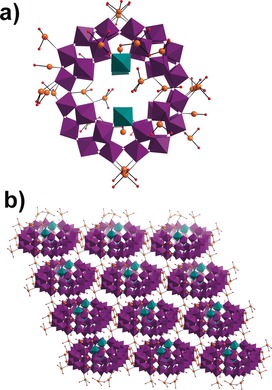
Structural representation: a) Compound (**2**) based on {P_8_W_48_} units. b) Connectivity of (**2**) in three dimensions. Color scheme: WO_6_ in {W_48_} units, purple polyhedra; WO_6_ {W_1_} growth points, teal polyhedra; Ag, orange spheres; oxygen, red spheres.

**Figure 3:**
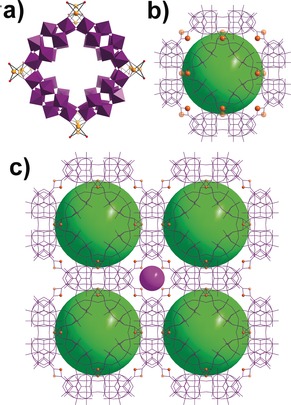
Structural representation of a) {P_8_W_48_}‐based building block in compound (**3**). b) Basic connectivity of the cuboctahedron. c) 3D cubic network in (**3**). Color scheme: orange spheres, Ag disordered over two sites on each bridging position. The two different pore types within the network are represented with a lime ball of 2.4 nm diameter, which outlines a spherical void of ≈4.8 nm^3^, and a purple ball of of 0.7 nm diameter, which outlines a spherical void of ≈0.2 nm^3^.

Structural control between the two distinct structures, (**1**) and (**2**), was achieved by varying reaction temperatures and the concentration of Ag^+^ ions, whilst maintaining the same pH 1.53. Compound (**1**) was obtained by heating at 80 °C for 30 minutes with a much higher concentration of Ag^+^ ions that replaces the K^+^ ions in the {P_8_W_48_} cavity of the starting materials and produces more {PW_9_} and {P_2_W_12_} fragments, which fully fill the growth sites on the {P_8_W_48_} base. This leads to the formation of molecular clusters {P_9_W_63_O_235_} and {P_10_W_66_O_251_} which co‐crystallize in compound (**1**). The synthesis of (**2**) under similar conditions but with a lower concentration of Ag^+^ ions and without heating yields some partially filled growth points but with no extended POM structure, such as {P_9_W_63_O_235_} and {P_10_W_66_O_251_} as in (**1**). Under such conditions Ag^+^ ions cannot substantially replace the K^+^ ions in the {P_8_W_48_} cavity of the starting materials and no significant amount of {PW_9_} and {P_2_W_12_} fragments are produced. Compound (**3**), synthesized with the reaction conditions of (**2**), but with a lower pH of 1.45, has no growth points filled with {W_1_} units. This is because lower pH is not favorable for {P_8_W_48_} to disassemble to produce fragments or even {W_1_} units. Also lower pH is not favorable for Ag^+^ ions to coordinate to oxo ligands because oxo has a higher affinity to protons. Therefore, fewer Ag^+^ ions are included in (**3**) and hence fewer O‐Ag‐O bridges are formed between the {P_8_W_48_} clusters, generating a less dense cubic framework compared with compounds (**1**) and (**2**) (Figure S5).

In conclusion, by varying the reaction conditions—the temperature, pH and the concentration of Ag^+^ ions—it is possible to control the synthesis of lacunary {PW_9_} and {P_2_W_12_} fragments which in turn produce high‐nuclearity tungstate clusters, {P_9_W_63_O_235_} and {P_10_W_66_O_251_}. Both clusters have the same {W_48_} base and roughly similarly sized {PW_9_} and {P_2_W_12_} additional units. These discoveries demonstrate that the {P_8_W_48_} cluster is an important building block for the construction of POM materials and networks utilizing cluster paneling, via extension from the eight possible growth points. The role of Ag^+^ is significant by filling the {P_8_W_48_} cavity to support the extended clusters, as further evidenced by the fact that Ag^+^ ions are needed to produce these clusters. For example, the potassium salt of the starting material's {P_8_W_48_} cluster forms readily from plenty of {P_2_W_12_} fragments, but further growth cannot be achieved without adding silver ions to replace K^+^ ions and support the key growth sites. Future work will focus on the reaction of Ag^+^ ions with other lacunary POM clusters as well as exploring the properties of the framework with regard to guest uptake and reactivity of the inner pores.

## Experimental Section

General experimental remarks: All chemicals were purchased from commercial sources and used without further purification. CCDC https://www.ccdc.cam.ac.uk/services/structures?id=doi:10.1002/anie.201911170 contain the supplementary crystallographic data for this paper. These data can be obtained free of charge from http://www.ccdc.cam.ac.uk/.

**Synthesis of Li_8_K_9.5_Ag_21_[H_16_P_10_W_66_O_251_]_0.5_[H_14_P_9_W_63_O_235_]_0.5_Cl_2_⋅50 H_2_O** (**1**): In a 25 mL round‐bottomed flask, LiNO_3_ (84 mg, 1.2 mmol) was dissolved in 12 mL of H_2_O, then K_28_Li_5_[H_7_P_8_W_48_O_184_]⋅92 H_2_O (102 mg, 6.9×10^−3^ mmol) was added and dissolved. The solution was adjusted to pH 1.53 by using HNO_3_ (70 %), then AgNO_3_ (35.7 mg, 0.21 mmol) was added. The mixture was then heated at 80 °C for 30 min then cooled down to room temperature. After two weeks well‐behaved block colorless crystals started to form in solution. The products were isolated after several weeks. The chlorine came from the trace amount in the starting materials. Yield: 0.06 g. Characteristic IR bands (in cm^−1^): ν_as_(H_2_O), 3439 (br),1620(m); ν_as_(Ag‐O), 1384 (s); ν_as_(P‐O), 1128 (s), 1086 (s); ν_as_(W‐O_t_), 1015 (w), 926 (br). Elemental analysis calcd (found) for Ag_21_Cl_2_H_115_Li_8_K_9.5_O_293_P_9.5_W_64.5_: Ag 11.5 (11.6), W 60.1 (60.3), K 1.88 (1.74), Li 0.28 (0.29). TGA water loss from room temperature to 400 °C, calcd (found) %: 4.6 (5.2).

**Synthesis of Li_8_K_13_Ag_13_[H_12_P_8_W_51_O_196_]⋅50 H_2_O** (**2**): In a 25 mL flask, LiNO_3_ (84 mg, 1.2 mmol) was dissolved in 12 mL of H_2_O, then K_28_Li_5_[H_7_P_8_W_48_O_184_]⋅92 H_2_O (102 mg, 6.9×10^−3^ mmol) was added. The solution was adjusted to pH 1.53 by using HNO_3_ (70 %), then AgNO_3_ (13.7 mg, 0.08 mmol) was added. The mixture was then stirred at room temperature for 5 min. After three weeks well‐behaved block, colorless crystals started to form in solution. These were isolated after several weeks. Yield: 0.12 g. Characteristic IR bands (in cm^−1^): ν_as_(H_2_O), 3439 (br), 1626(m); ν_as_(Ag‐O), 1383 (s); ν_as_(P‐O), 1140 (s), 1075 (s); ν_as_(W‐O_t_), 1015 (w), 926 (br). Elemental analysis calcd (found) for H_100_O_238_Li_12_K_13_Ag_11_P_8_W_50_: Ag 8.97 (7.84), W 60.0 (58.7), K 3.25 (3.65), Li 0.35 (0.29). TGA water loss from room temperature to 400 °C, calcd (found) %: 5.8 (6.5).

**Synthesis of Li_10_K_12_Ag_4_[H_14_P_8_W_48_O_184_]⋅170 H_2_O** (**3**): In a 25 mL flask, LiNO_3_ (84 mg, 1.2 mmol) was dissolved in 12 mL of H_2_O, then K_28_Li_5_[H_7_P_8_W_48_O_184_]⋅92 H_2_O (102 mg, 6.9×10^−3^ mmol) was added. The solution was adjusted to pH 1.45 by using HNO_3_ (75 %), then AgNO_3_ (16.5 mg, 0.1 mmol) was added. The mixture was then stirred at room temperature for 5 min. After three weeks well‐behaved block colorless crystals started to form in solution. These were isolated after several weeks. Yield: 0.06 g. Characteristic IR bands (in cm^−1^): ν_as_(H_2_O), 3416 (br), 1626(m); ν_as_(Ag‐O), 1412 (w); ν_as_(P‐O), 1134 (s), 1081 (s); ν_as_(W‐O_t_), 1015 (w), 926 (br). Elemental analysis calcd (found) for Ag_4_H_354_Li_10_K_12_O_354_P_8_W_48_: Ag 2.7 (2.9), W 54.9 (55.1), K 2.92 (3.17), Li 0.43 (0.43). TGA water loss from room temperature to 400 °C, calcd (found) %: 19.0 (19.5).

## Conflict of interest

The authors declare no conflict of interest.

## Supporting information

As a service to our authors and readers, this journal provides supporting information supplied by the authors. Such materials are peer reviewed and may be re‐organized for online delivery, but are not copy‐edited or typeset. Technical support issues arising from supporting information (other than missing files) should be addressed to the authors.

Supplementary
