# Supplementary material for: Controlling the Reactivity of the [P8W48O184]40− Inorganic Ring and Its Assembly into POMZite Inorganic Frameworks with Silver Ions
Source: Angew Chem Int Ed Engl. 2019 Oct 17;58(48):17282–6. doi: 10.1002/anie.201911170 (PMC6900112; doi:10.1002/anie.201911170)
Supplement: Supplementary file 1 — Supplementary [file ANIE-58-17282-s001.pdf]

Angewandte Chemie
Eine Zeitschrift der Gesellschaft Deutscher Chemiker

Supporting Information

Controlling the Reactivity of the $[P_8W_{48}O_{184}]^{40-}$ Inorganic Ring and Its Assembly into POMZite Inorganic Frameworks with Silver Ions

Cai-Hong Zhan, Qi Zheng, De-Liang Long, Laia Vilà-Nadal, and Leroy Cronin**

anie_201911170_sm_miscellaneous_information.pdf

1. General Experimental Section

Inductively Coupled Plasma Optical Emission Spectroscopy (ICP-OES): A minimum of 10 mg of each compound was submitted to the *Institut für Festkörperforschung* in Jülich, Germany, for analysis. Samples were digested in a 1:1 mixture of HNO_3 and H_2O_2. A *TJA-IRIS-Advantage* spectrometer with echelle optics and CID semiconductor was used to observe across a wavelength range of 170 – 900 nm.

Microanalysis: Carbon, hydrogen and nitrogen contents were determined using an *EA 1110 CHNS, CE-440 Elemental Analyser*. 5 – 10 mg samples of the relevant compounds were submitted to the *Microanalytical Service* within the *School of Chemistry*, *University of Glasgow*..

Fourier Transform Infrared Spectroscopy (FTIR): The materials were prepared as KBr pellets and FTIR spectra were collected in transmission mode using a JASCO FT/IR 4100 spectrometer. Wavenumbers (n) are given in cm^{-1}; intensities are denoted as w = weak, m = medium, br = broad, s = strong.

Powder X-Ray Diffraction (XRD): Powder XRD patterns were collected on Oxford Diffraction Gemini Ultra diffractometer (λ(CuKα) = 1.5405 Å) at room temperature.

^{31}P-Nuclear Magnetic Resonance Spectroscopy (^{31}P-NMR): ^{31}P-NMR spectroscopy was performed on a Bruker DPX 400 or Avance 400 spectrometer (400 MHz), with chemical shift (d) referenced to H_3PO_4 at 0.00 ppm. Generally, ~20 mg of sample was dissolved in 1 mL of 9:1 1 M LiCl:H_2O, before transferring to an NMR tube.

UV-Visible Spectroscopy: The UV-Visible spectra of the as-synthesized compound **1**-**3** in water were collected on UV-Visible spectrophotometer (Cary 4000, Agilent, USA).

2. Synthesis and Experimental Section

Synthesis of $Li_8K_{9.5}Ag_{21}[H_{16}P_{10}W_{66}O_{251}]_{0.5}[H_{14}P_9W_{63}O_{235}]_{0.5}Cl_2\cdot 50H_2O$ (1)

In a 25 mL round bottomed flask, $LiNO_3$ (84 mg, 1.2 mmol) was dissolved in 12 mL of H_2O, then $K_{28}Li_5[H_7P_8W_{48}O_{184}]\cdot 92H_2O$ (102 mg, 6.9 x 10^{-3} mmol) was added and dissolved. The solution was adjusted to pH = 1.53 by using HNO_3 (70%), then $AgNO_3$ (35.7 mg, 0.21 mmol) was added. The mixture was then heated at 80˚C for 30 min then cooled down to room temperature. After two weeks well behaved block colorless crystals start to form in solution. The products are isolated in several weeks. The chlorine came from the trace amount in the starting materials. Yield: 0.06 g. Characteristic I.R. bands (in cm^{-1}): $\nu_{as}(H_2O)$, 3439 (br),1620(m); ν_{as}(Ag–O), 1384 (s); ν_{as}(P–O), 1128 (s), 1086 (s); $\nu_{as}(W–O_t)$, 1015 (w), 926 (br). Elemental analysis calcd. (found) for $Ag_{21}Cl_2H_{115}Li_8K_{9.5}O_{293}P_{9.5}W_{64.5}$: Ag 11.5 (11.6), W 60.1 (60.3), K 1.88 (1.74), Li 0.28 (0.29).

Synthesis of $Li_8K_{13}Ag_{13}[H_{12}P_8W_{51}O_{196}]\cdot 50H_2O$ (2)

In a 25 mL flask, $LiNO_3$ (84 mg, 1.2 mmol) was dissolved in 12 mL of H_2O, then $K_{28}Li_5[H_7P_8W_{48}O_{184}]\cdot 92H_2O$ (102 mg, 6.9 x 10^{-3} mmol) was added. The solution was adjusted to pH = 1.53 by using HNO_3 (70%), then

$AgNO_3$ (13.7 mg, 0.08 mmol) was added. The mixture was then stirred at room temperature for 5 min. After three weeks well behaved block, colorless crystals start to form in solution. These are isolated in several weeks. Yield: 0.12 g. Characteristic I.R. bands (in cm^{-1}): $\nu_{as}(H_2O)$, 3439 (br), 1626(m); ν_{as}(Ag–O), 1383 (s); ν_{as}(P–O), 1140 (s), 1075 (s); $\nu_{as}(W–O_t)$, 1015 (w), 926 (br). Elemental analysis calcd. (found) for $H_{100}O_{238}Li_{12}K_{13}Ag_{11}P_8W_{50}$: Ag 8.97 (7.84), W 60.0 (58.7), K 3.25 (3.65), Li 0.35 (0.29).

Synthesis of $Li_{10}K_{12}Ag_4[H_{14}P_8W_{48}O_{184}]\cdot 170H_2O$ (**3**)

In a 25 mL flask, $LiNO_3$ (84 mg, 1.2 mmol) was dissolved in 12 mL of H_2O, then $K_{28}Li_5[H_7P_8W_{48}O_{184}]\cdot 92H_2O$ (102 mg, 6.9 x 10^{-3} mmol) was added. The solution was adjusted to pH = 1.45 by using HNO_3 (75%), then $AgNO_3$ (16.5 mg, 0.1 mmol) was added. The mixture was then stirred at room temperature for 5 min. After three weeks well behaved block colorless crystals start to form in solution. These are isolated in several weeks. Yield: 0.06 g. Characteristic I.R. bands (in cm^{-1}): $\nu_{as}(H_2O)$, 3416 (br), 1626(m); ν_{as}(Ag–O), 1412 (w); ν_{as}(P–O), 1134 (s), 1081 (s); $\nu_{as}(W–O_t)$, 1015 (w), 926 (br). Elemental analysis calcd. (found) for $Ag_4H_{354}Li_{10}K_{12}O_{354}P_8W_{48}$: Ag 2.7 (2.9), W 54.9 (55.1), K 2.92 (3.17), Li 0.43 (0.43).

3. Crystal structure determinations

Suitable single crystal was selected and mounted onto the end of a thin glass fiber using Fomblin oil. X-ray diffraction intensity data were measured at 150(2) K on a Bruker Apex II Quasar diffractometer ($\lambda(Mo_{K\alpha})$ = 0.71073 Å). Corrections for incident and diffracted beam absorption effects were applied using either empirical or analytical methods respectively,[1,2] while data reduction was performed using either the Apex2 software as supplied by the manufacturers. Final structure solution and refinement were carried out with SHELXS-97 and SHELXL-2018 (or later versions) *via* the WinGX software suite,[3,4] with all structures solved by direct methods and refined using a full matrix least squares on F^2 method. Selected details of the data collection and structural refinement of each compound can be found in Table S1-S3, and full details are available in the corresponding CIF files. The X-ray crystallographic data for structures reported in this article have been deposited at the Cambridge Crystallographic Data Centre, under deposition number CCDC 1892362-1892364. These data can be obtained free of charge via www.ccdc.cam.ac.uk/data_request/cif, or by emailing data_request@ccdc.cam.ac.uk, or by contacting The Cambridge Crystallographic Data Centre, 12 Union Road, Cambridge CB2 1EZ, UK; fax: +44 1223 336033.

In the structure determination process, the structures were solved by using direct methods and tungsten sites were firstly identified on the positions of heaviest electron density peaks. Then light oxo ligands around tungsten sites were recognized and define the polyoxotungstate frameworks. Ag and K positions were subsequently found from heavy electron density peaks outside the POM frameworks using the criteria Ag-O distances being about 2.4 Å and K-O distances being about 2.7 Å. The occupancies of Ag and K were initially refined and then fixed to reasonable values. Water ligands around the Ag and K sites were then defined by checking their coordination spheres.

(1) G. Sheldrick, *ActaCrystallographica Section A*, 1990, **46**, 467-473.
(2) G. Sheldrick, *ActaCrystallographica Section A*, 2008, **64**, 112-122.
(3) L. Farrugia, *J. Appl. Crystallogr.*, 1999, **32**, 837-838.
(4) R. C. Clark, J. S. Reid, *ActaCrystallogr., Sect. A*, 1995, **51**, 887-897.

Table S1. Crystal data and structure refinement for **1**.

Identification code	**1**	
Empirical formula	Ag21 H115 Cl2 K9.5 Li8 O293 P9.5 W64.5	
Formula weight	19719.6	
Temperature	150(2) K	
Wavelength	0.71073 Å	
Crystal system	Monoclinic	
Space group	P 21/m	
Unit cell dimensions	a = 21.3544(5) Å	α = 90°.
	b = 25.5596(6) Å	β = 98.4280(10)°.
	c = 29.5791(7) Å	γ = 90°.
Volume	15970.2(7) $Å^3$	
Z	2	
Density (calculated)	4.101 Mg/m^3	
Absorption coefficient	24.665 mm^{-1}	
F(000)	17200	
Crystal size	0.100 x 0.100 x 0.030 mm^3	
Theta range for data collection	1.251 to 26.000°.	
Index ranges	-25<=h<=26, -31<=k<=20, -36<=l<=36	
Reflections collected	126880	
Independent reflections	32116 [R(int) = 0.079]	
Completeness to theta = 25.000°	100.0 %	
Refinement method	Full-matrix least-squares on F^2	
Data / restraints / parameters	32116 / 1200 / 1992	
Goodness-of-fit on F^2	1.062	
Final R indices [I>2sigma(I)]	R1 = 0.0569, wR2 = 0.1304	
R indices (all data)	R1 = 0.0886, wR2 = 0.1462	
Extinction coefficient	n/a	
Largest diff. peak and hole	3.87 and -3.22 e.$Å^{-3}$	

Table S2. Crystal data and structure refinement for **2**.

Identification code	**2**	
Empirical formula	Ag13 H112 K13 Li8 O246 P8 W51	
Formula weight	15639.13	
Temperature	150(2) K	
Wavelength	0.71073 Å	
Crystal system	Triclinic	
Space group	P -1	
Unit cell dimensions	a = 15.0945(8) Å	α = 112.816(3)°.
	b = 21.6623(11) Å	β = 106.437(4)°.
	c = 22.0017(12) Å	γ = 92.485(4)°.
Volume	6264.2(6) $Å^3$	
Z	1	
Density (calculated)	4.146 Mg/m^3	
Absorption coefficient	24.672 mm^{-1}	
F(000)	6856	
Crystal size	0.115 x 0.091 x 0.073 mm^3	
Theta range for data collection	1.913 to 25.080°.	
Index ranges	-18<=h<=17, -25<=k<=21, -26<=l<=26	
Reflections collected	84556	
Independent reflections	22124 [R(int) = 0.148]	
Completeness to theta = 25.000°	100.0 %	
Refinement method	Full-matrix least-squares on F^2	
Data / restraints / parameters	22124 / 823 / 1481	
Goodness-of-fit on F^2	1.012	
Final R indices [I>2sigma(I)]	R1 = 0.0662, wR2 = 0.1351	
R indices (all data)	R1 = 0.1464, wR2 = 0.1718	
Extinction coefficient	n/a	
Largest diff. peak and hole	2.78 and -2.69 e.$Å^{-3}$	

Table **S3**. Crystal data and structure refinement for **3**.

Identification code	**3**	
Empirical formula	Ag4 H354 K12 Li10 O354 P8 W48	
Formula weight	16063.45	
Temperature	150(2) K	
Wavelength	0.71073 Å	
Crystal system	Monoclinic	
Space group	Pm-3m	
Unit cell dimensions	a = 29.8933(9) Å	α = 90°.
	b = 29.8933(9) Å	β = 90°.
	c = 29.8933(9) Å	γ = 90°.
Volume	26713(2) $Å^3$	
Z	3	
Density (calculated)	2.996 Mg/m^3	
Absorption coefficient	15.941 mm^{-1}	
F(000)	21912	
Crystal size	0.100 x 0.100 x 0.050 mm^3	
Theta range for data collection	1.18 to 25.76°.	
Index ranges	-36<=h<=36, -17<=k<=36, -35<=l<=36	
Reflections collected	202724	
Independent reflections	4912 [R(int) = 0.079]	
Completeness to theta = 26.00°	99.9 %	
Refinement method	Full-matrix least-squares on F^2	
Data / restraints / parameters	4912 / 0 / 223	
Goodness-of-fit on F^2	1.334	
Final R indices [I>2sigma(I)]	R1 = 0.0718, wR2 = 0.1755	
R indices (all data)	R1 = 0.1399, wR2 = 0.2801	
Largest diff. peak and hole	2.52 and -1.57 e.$Å^{-3}$	

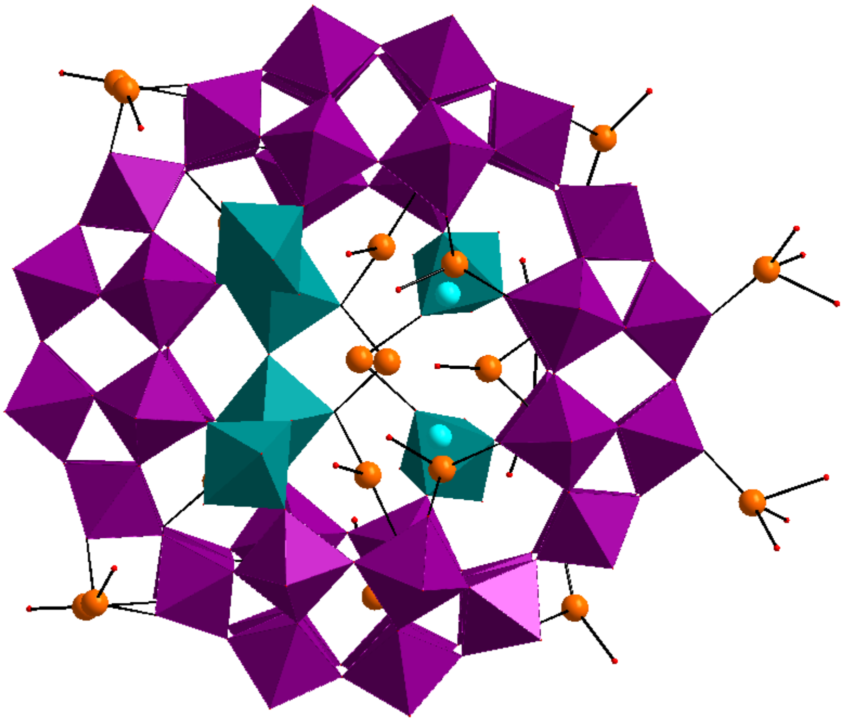

Figure S1. A close bottom view of the Ag^{+} ions located inside and outside the $\{P_{10}W_{66}\}_{0.5}\{P_9W_{63}\}_{0.5}$ cluster determined in **1**. Colour Scheme: W purple and teal polyhedrons; Ag orange spheres; O red spheres; Cl turquoise spheres. The $\{PW_9\}$ and $\{P_2W_{12}\}$ add-on units are omitted for clearity.

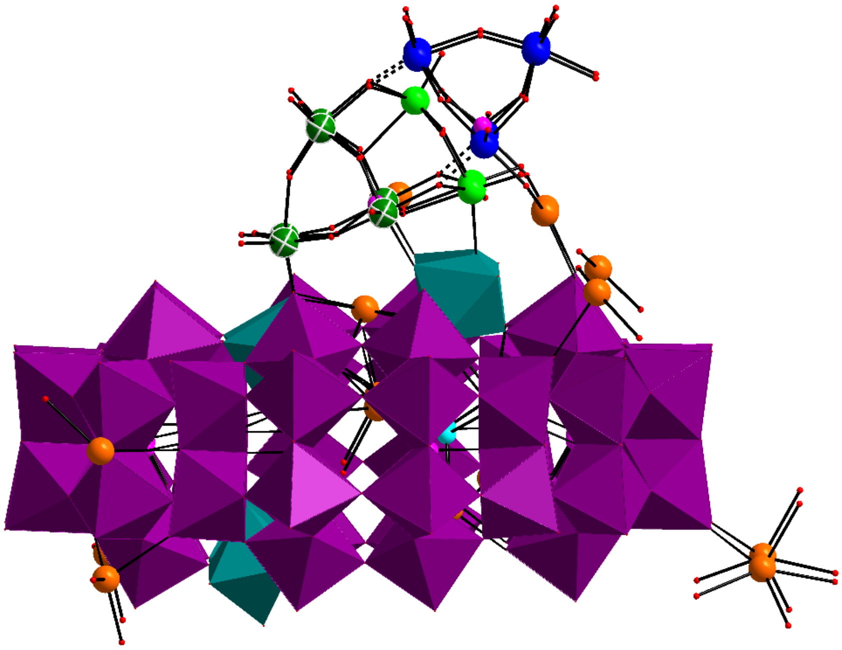

Figure S2. A close side view of the disorder model of the $\{PW_9\}$ and $\{P_2W_{12}\}$ add-on units and Ag^{+} ions located inside and outside the $\{P_{10}W_{66}\}_{0.5}\{P_9W_{63}\}_{0.5}$ cluster determined in **1**. Colour Scheme: W, purple and teal polyhedrons or blue and green spheres; Ag, orange spheres; O, red spheres; Cl, turquoise spheres. The six front ellipse green colored W sites are in full occupancy and are shared by the $\{PW_9\}$ and $\{P_2W_{12}\}$ add-on units. The remaining light green colored W sites belong to $\{PW_9\}$, while the remaining blue colored W sites belong to $\{P_2W_{12}\}$.

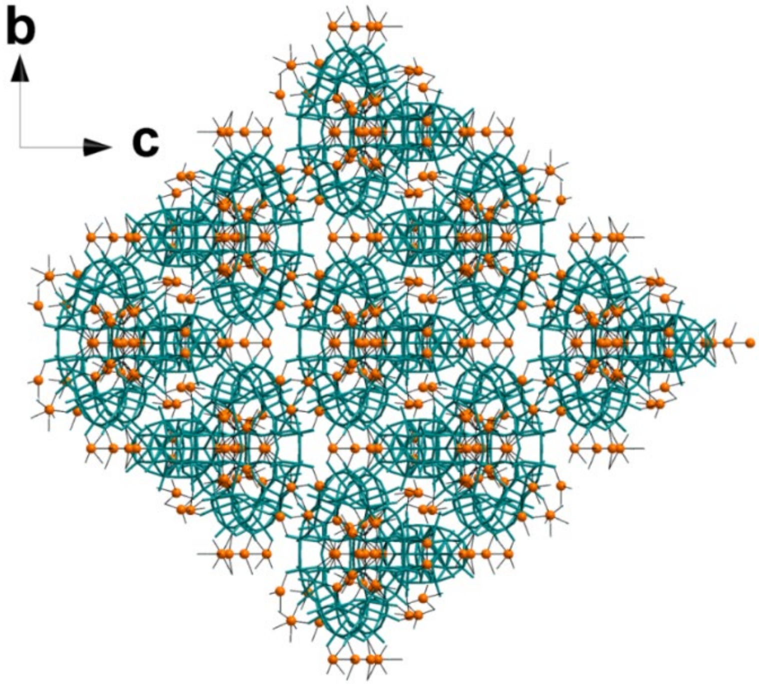

Figure S3. Representation of the 3D framework linked by Ag^+ ions in **1**. The Ag atoms are represented as orange spheres. The $\{P_9W_{63}O_{235}\}$ and $\{P_{10}W_{66}O_{251}\}$ clusters are overlapped and are represented in teal wireframe mode.

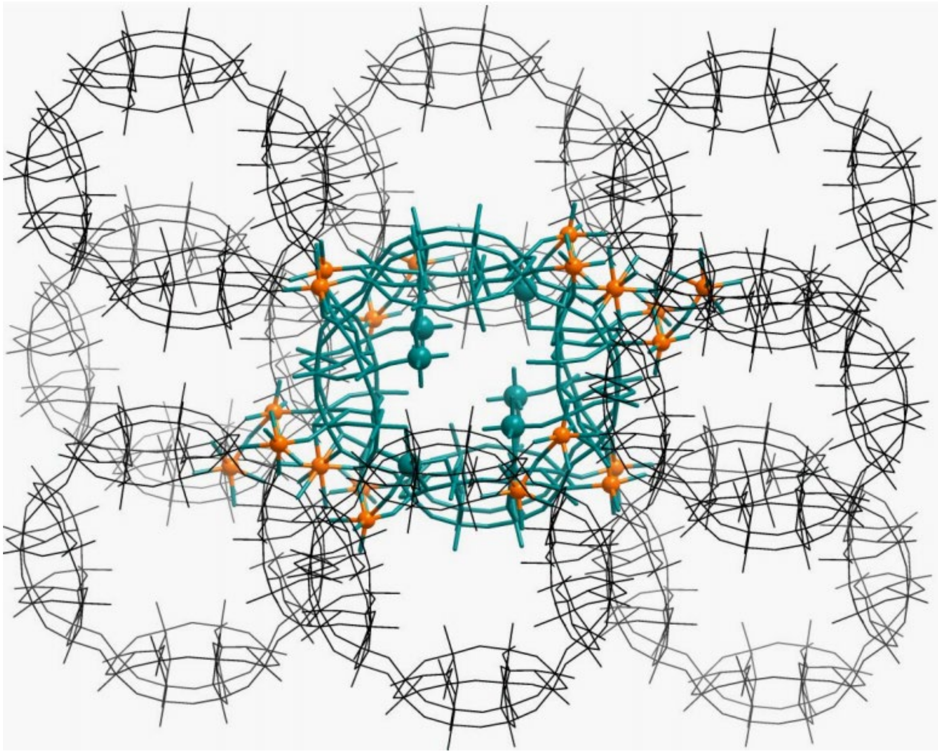

Figure S4. Molecule packing mode observed in **2**. The Ag atoms are represented as orange spheres. The central $\{P_8W_{48}\}$ cluster is shown in teal stick mode (except the add-on positions, on which W atoms are shown as balls). For clarity, only the Ag atoms around teal colored $\{P_8W_{48}\}$ cluster are shown and the eight adjacent clusters are represented in black wireframe modes.

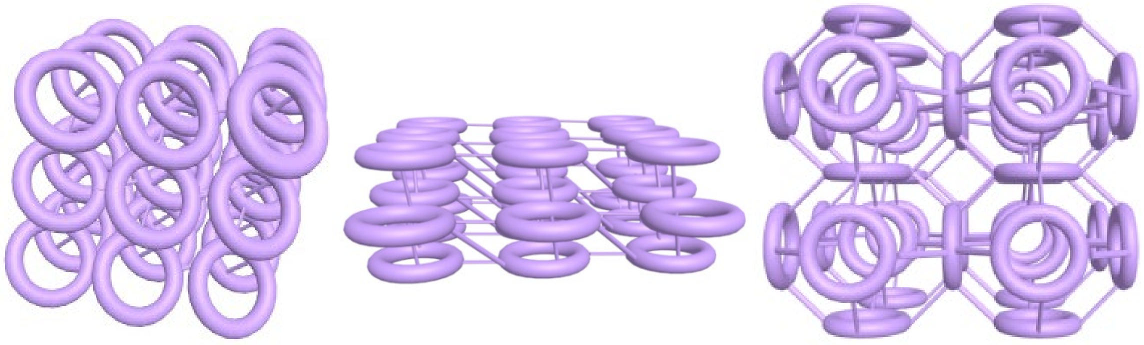

(a) (b) (c)

Figure S5. Representative packing modes of the $\{W_{48}\}$ rings in compound **1** (chain), **2** (column) and **3** (cube) shows cavities and channels inside networks. Major O-Ag-O links are shown to illustrate the topologies of the networks.

4. Fourier Transform Infrared Spectroscopy

Of particular interest here are the obvious and apparent signals stretching between 500 and 800 cm^{-1}. Tungstate POMs characteristically exhibit this signal as it corresponds to the W-O-W bridges generally present in these structures. The peak around 926 cm^{-1} is also characteristic of tungstate POMs and corresponds to terminal W=O bonds. Beyond these characteristic stretching frequencies common to many POMs, it becomes very difficult to meaningfully assign specific signals to specific parts of the structure due to the highly connected nature of these molecules altering the degrees of freedom available to individual atoms and groups in ways that are not easy to predict. However, there is an obvious peak around 1384 cm^{-1} which corresponds to the stretching of the Ag-O bonds. One more signal that could feasibly be assigned is around 3422 cm^{-1}, which is the characteristic of O-H bonds of water molecules.

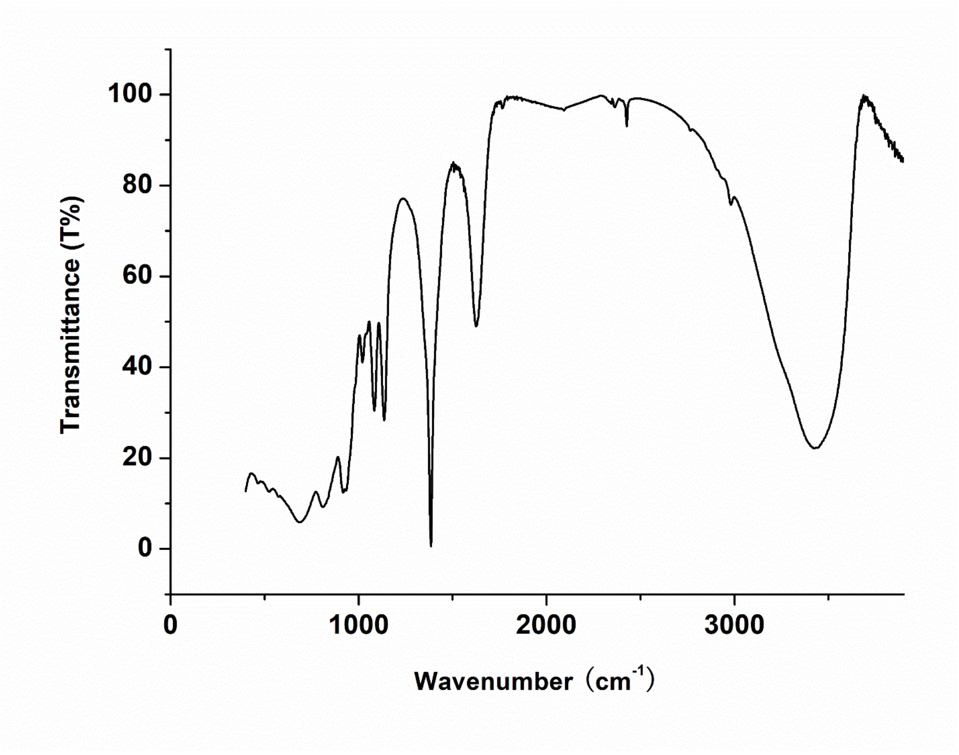

Figure S6. FT-IR spectra of compound **1**.

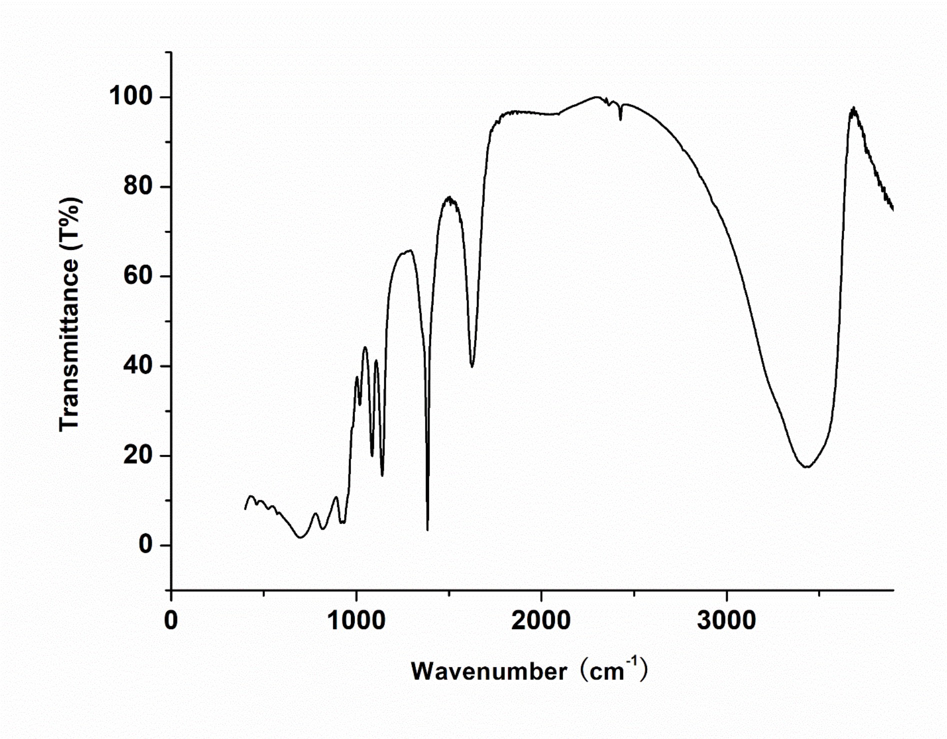

Figure S7. FT-IR spectra of compound **2**.

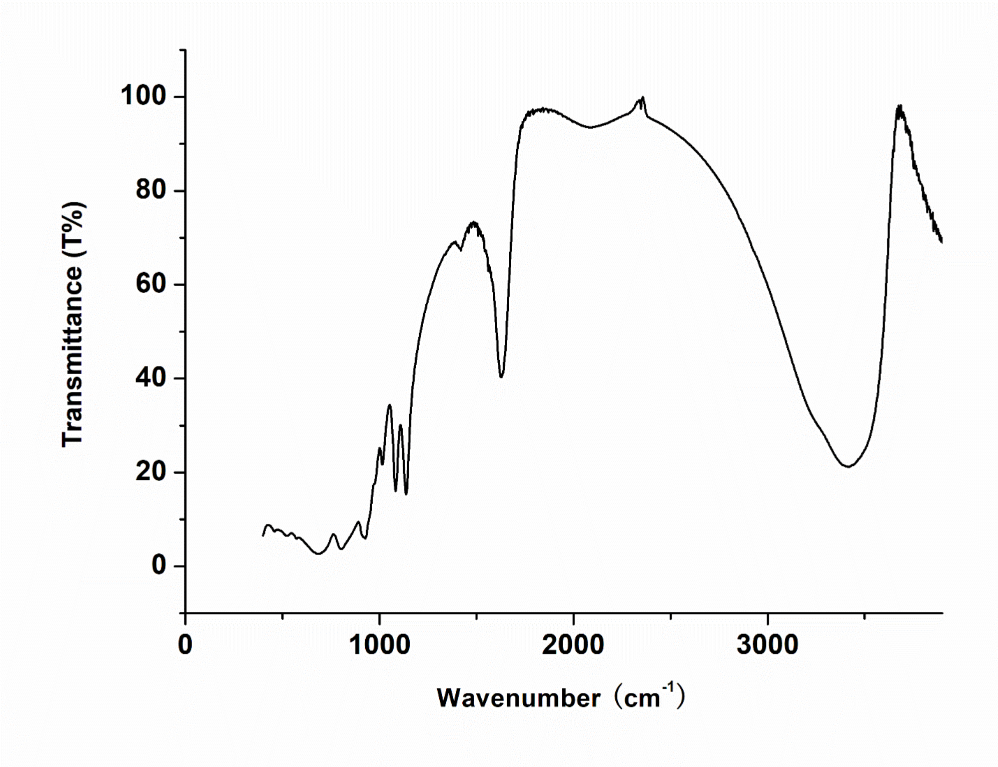

Figure S8. FT-IR spectra of compound **3**.

5. Powder X-Ray Diffraction

To check the purity of **1-3**, Powder X-Ray Diffraction (PXRD) was conducted. The experimental and simulated PXRD patterns are shown in Figure S12-14. Their peak positions are in good agreement with each other, indicating the phase purity of the product. The differences in intensity may be due to the preferred orientation of the powder sample.

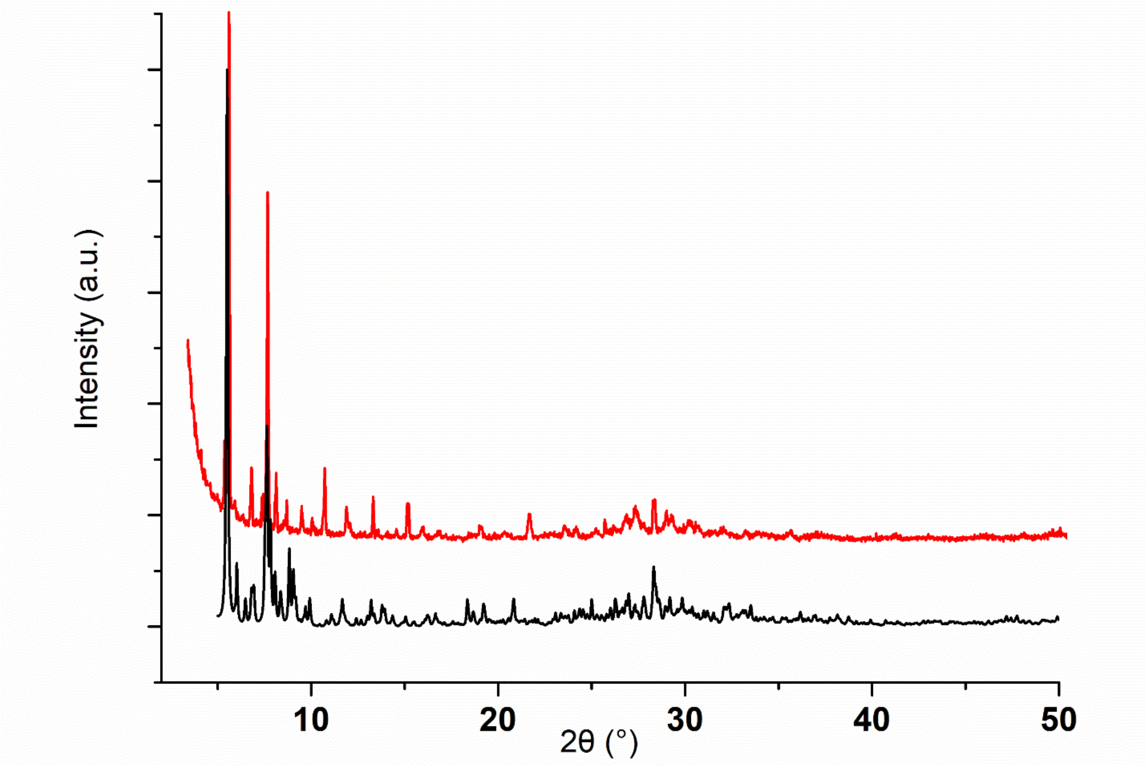

Figure S9. Experimental (red) and simulated (black) PXRD patterns of compound **1**.

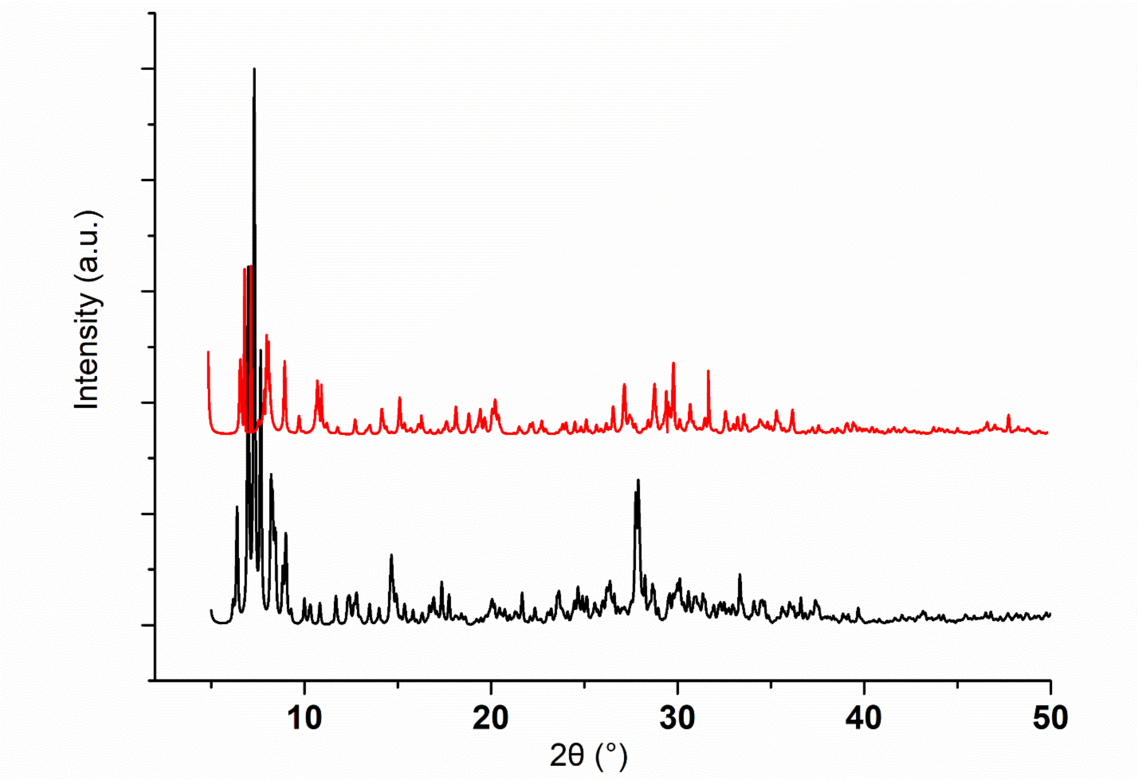

Figure S10. Experimental (red) and simulated (black) PXRD patterns of compound **2**.

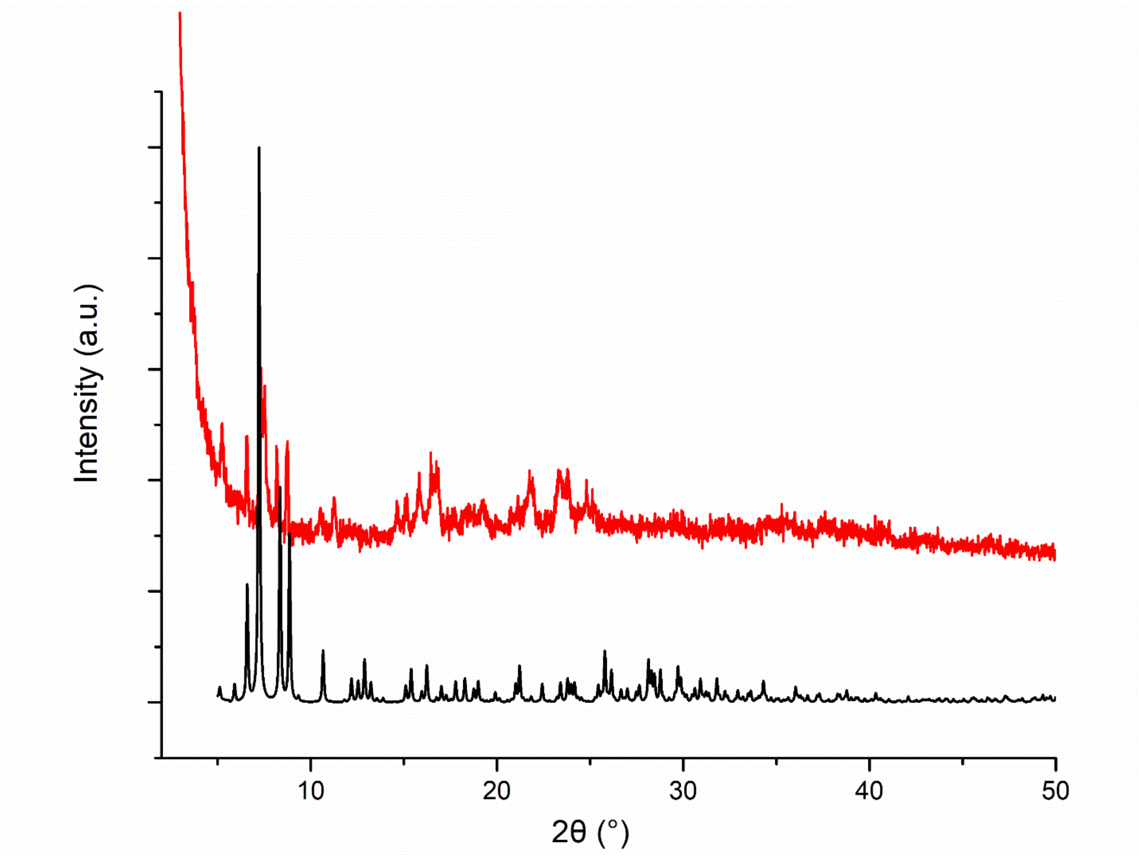

Figure S11. Experimental (red) and simulated (black) PXRD patterns of compound **3**.

6. ^{31}P-Nuclear Magnetic Resonance Spectroscopy

^{31}P NMR spectrum of compound **1** in lithium chloride aqueous solution exhibits six peaks at 3.62, 1.75, -8.81, -10.72, -12.21 and -14.06 ppm. It corresponds to a new species with inequivalent phosphorus atoms from different building blocks ($[H_{16}P_{10}W_{66}O_{251}]$ and $[H_{12}P_9W_{63}O_{235}]$). The spectrum of compound **2** in lithium chloride aqueous solution exhibits two peaks at -0.77 and -13.98 ppm. It corresponds to a new species with inequivalent phosphorus atoms due to partially filling of the {W1} growth positions in the solution. The spectrum of compound **3** in lithium chloride aqueous solution exhibits only one peak at -7.13 ppm. It corresponds to the equivalent phosphorus atoms in $\{P_8W_{48}\}$ in the solution.

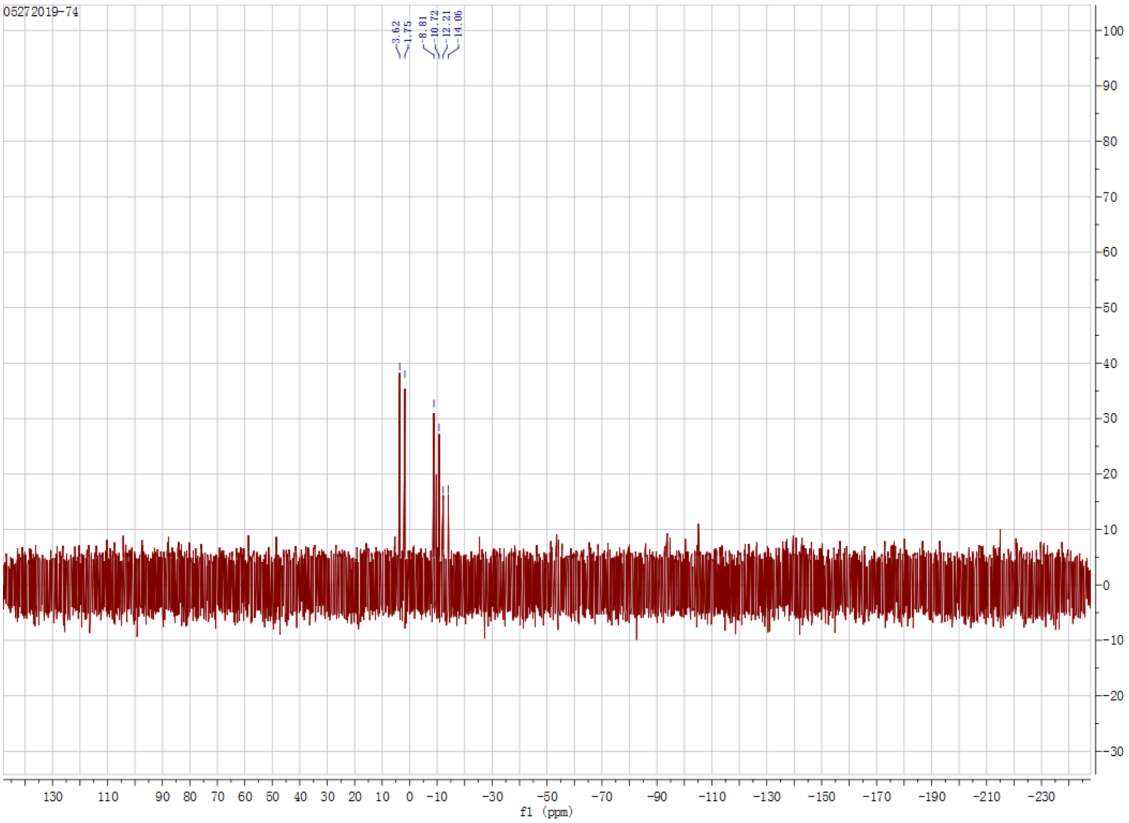

Figure S12. ^{31}P NMR spectra in lithium chloride H_20 solutions of compound **1**.

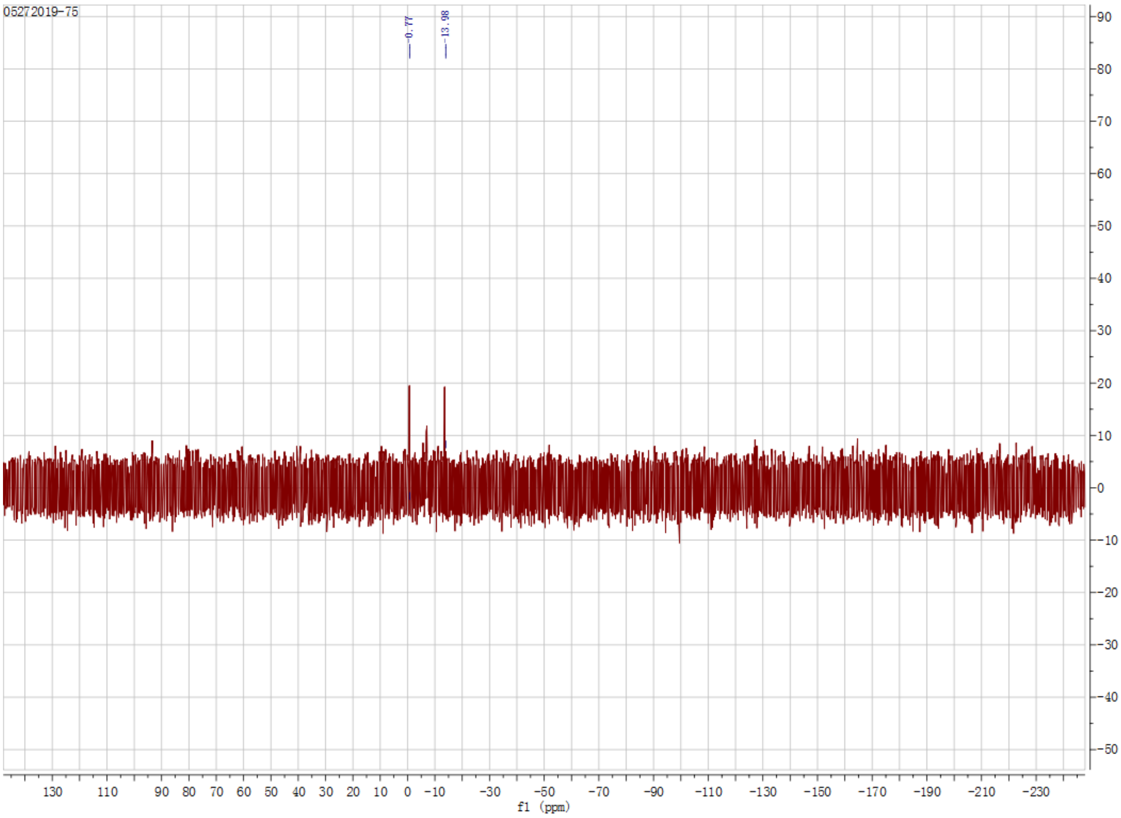

Figure S13. ^{31}P NMR spectra in lithium chloride H_2O solutions of compound **2**.

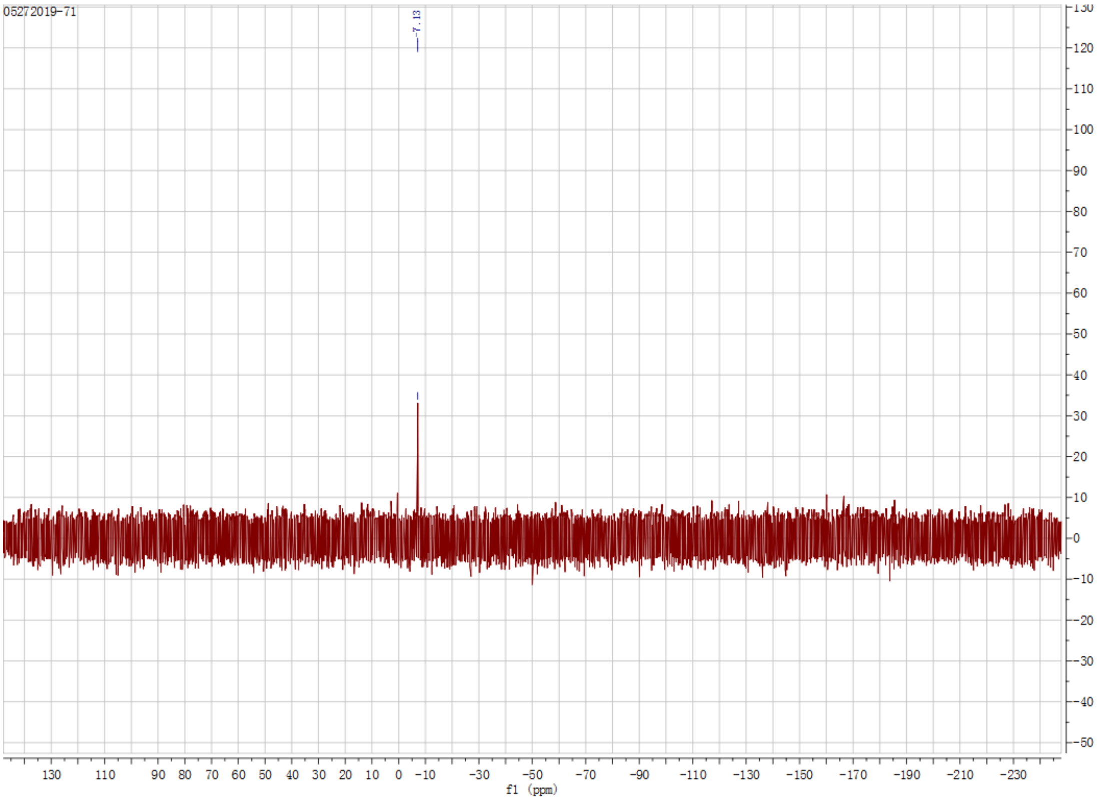

Figure S14. ^{31}P NMR spectra in lithium chloride H_2O solutions of compound **3**.

7. UV-Visible spectra

The UV-Visible spectra of the as-synthesized compound **1**-**3** in water shows no absorption band in the range of 350–800 nm.

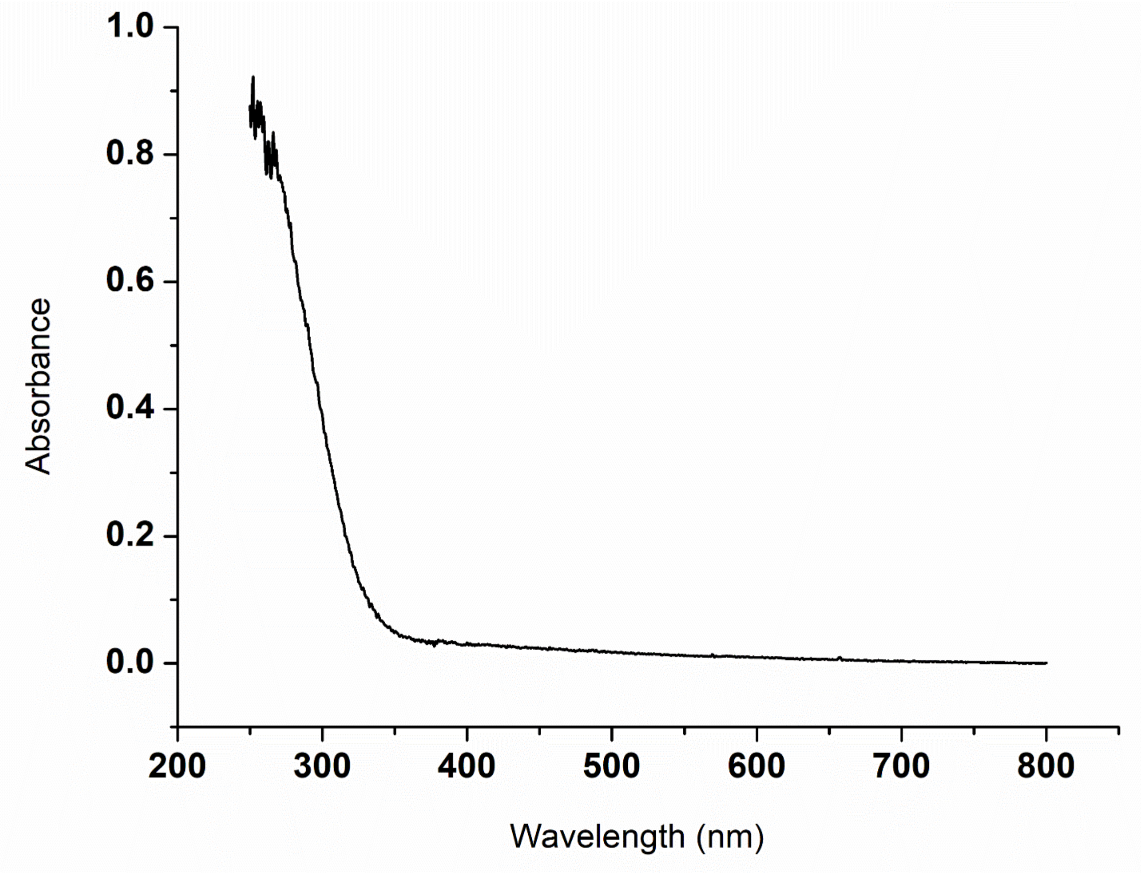

Figure S15. The UV-Visible spectra of the compound **1**.

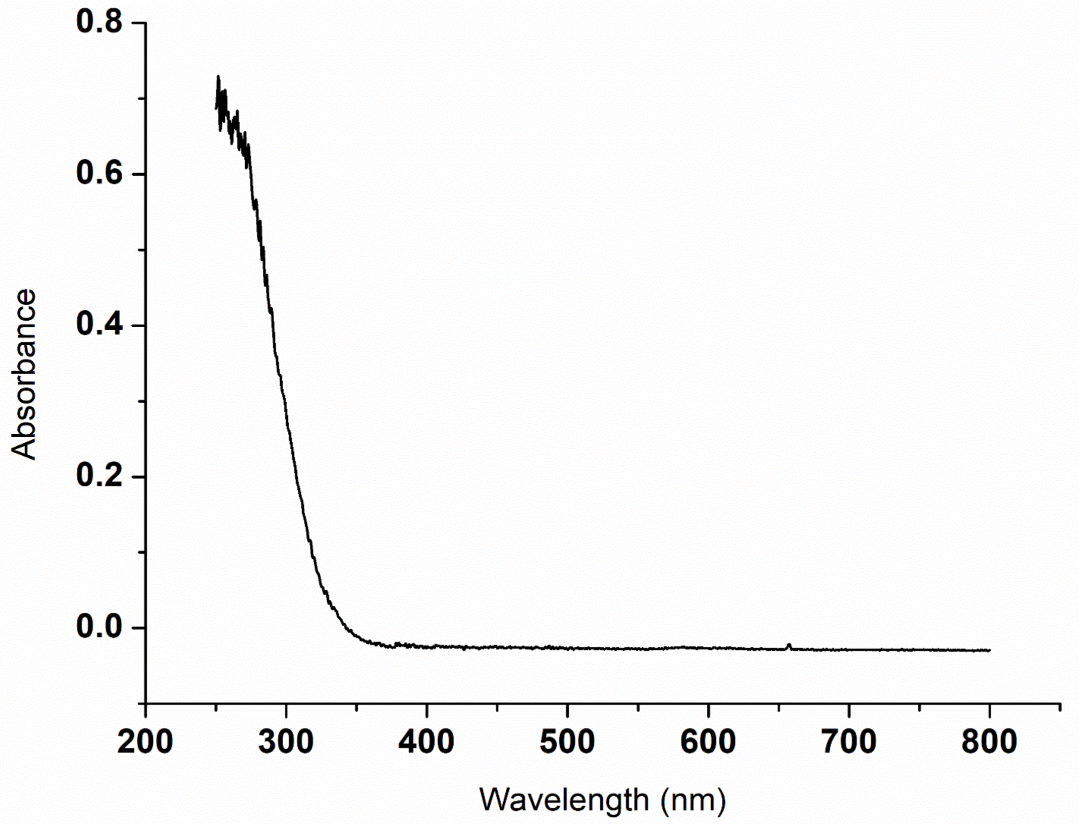

Figure S16. The UV-Visible spectra of the compound **2**.

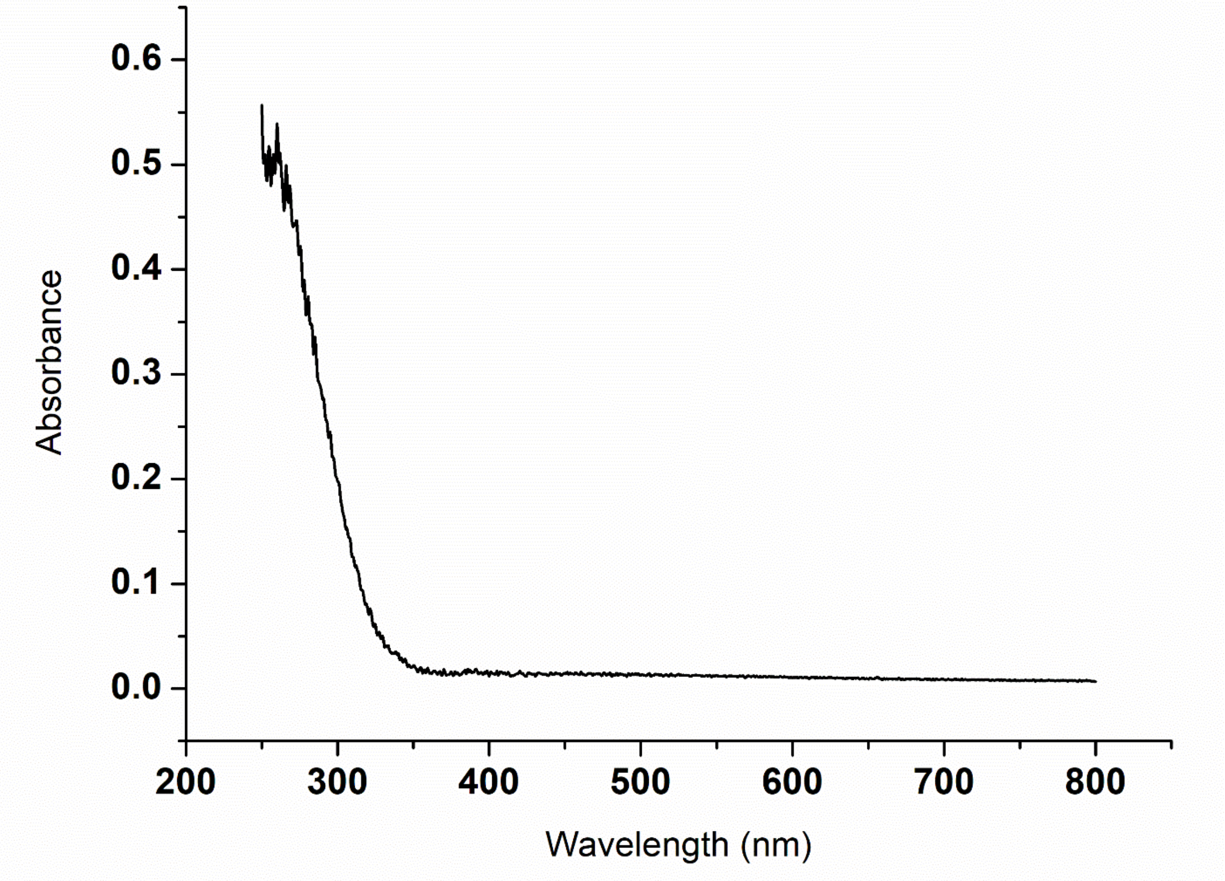

Figure S17. The UV-Visible spectra of the compound **3**.
